# IDOD-YOLOV7: Image-Dehazing YOLOV7 for Object Detection in Low-Light Foggy Traffic Environments

**DOI:** 10.3390/s23031347

**Published:** 2023-01-25

**Authors:** Yongsheng Qiu, Yuanyao Lu, Yuantao Wang, Haiyang Jiang

**Affiliations:** 1 School of Electrical and Control Engineering, North China University of Technology, Beijing 100144, China; 2 School of Information Science and Technology, North China University of Technology, Beijing 100144, China

**Keywords:** IDOD-YOLOV7, object detection, low-light foggy images, autonomous driving

## Abstract

Convolutional neural network (CNN)-based autonomous driving object detection algorithms have excellent detection results on conventional datasets, but the detector performance can be severely degraded in low-light foggy weather environments. Existing methods have difficulty in achieving a balance between low-light image enhancement and object detection. To alleviate this problem, this paper proposes a foggy traffic environment object detection framework, IDOD-YOLOV7. This network is based on joint optimal learning of image defogging module IDOD (AOD + SAIP) and YOLOV7 detection modules. Specifically, for low-light foggy images, we propose to improve the image quality by joint optimization of image defogging (AOD) and image enhancement (SAIP), where the parameters of the SAIP module are predicted by a miniature CNN network and the AOD module performs image defogging by optimizing the atmospheric scattering model. The experimental results show that the IDOD module not only improves the image defogging quality for low-light fog images but also achieves better results in objective evaluation indexes such as PSNR and SSIM. The IDOD and YOLOV7 learn jointly in an end-to-end manner so that object detection can be performed while image enhancement is executed in a weakly supervised manner. Finally, a low-light fogged traffic image dataset (FTOD) was built by physical fogging in order to solve the domain transfer problem. The training of IDOD-YOLOV7 network by a real dataset (FTOD) improves the robustness of the model. We performed various experiments to visually and quantitatively compare our method with several state-of-the-art methods to demonstrate its superiority over the others. The IDOD-YOLOV7 algorithm not only suppresses the artifacts of low-light fog images and improves the visual effect of images but also improves the perception of autonomous driving in low-light foggy environments.

## 1. Introduction

Environment perception, behavior decision, and motion control are the three major tasks of autonomous driving, for which object detection is an important component of environment perception. However, the accuracy and real-time performance of sensor acquisition data will be seriously affected in severe weather environments. For example, in low-light foggy environments, this leads to image degradation problems that can seriously affect the object detection performance of autonomous vehicles [1].

**Figure 1:**
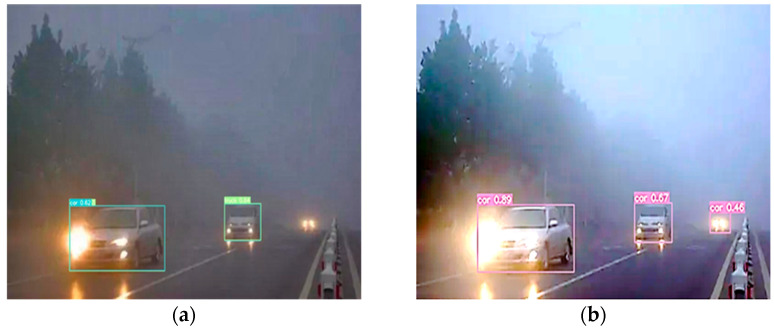
Object Detection in Low-Light Foggy Traffic Environments. (**a**) Image visualization and detection accuracy are low for object detection using only YOLOV7, YOLOV7; (**b**) The method proposed in this paper not only improves the detection accuracy but also reduces the leakage rate, IDOD-YOLOV7 (Ours).

Most of the mainstream detection algorithms, such as Faster R-CNN [2], SSD [3], RetinaNet [4], YOLO [5], etc., have good performance when the object detection is performed in a good-weather road environment. However, if the aforementioned object detection methods are utilized directly for object identification in adverse weather, particularly in environments with low-light fog, the detection performance would be severely compromised [6]. An example of object detection in a low-light foggy traffic environment is shown in Figure 1, where if the image enhancement process is applied to the low-light fog image, the potential information of the image can be further recovered and the accuracy of object detection can be improved. 

To address this problem, Cao J et al. [7] used a weighted mask and loss function enhancement to improve the SSD model, which effectively reduced the vehicle miss detection due to the lack of local information, thus improving the accuracy of small object vehicle detection in severe weather such as fog, but with slow detection speed. Han X [8] proposed an enhanced RCNN vehicle detection model that incorporates rich location and background information and reduces information loss caused by feature mapping in downsampling, but suffers from omissions and false detection in foggy environments. Huang et al. [9] proposed to use two sub-networks for joint learning of image enhancement and object detection to reduce the effect of image degradation by sharing the feature extraction layer; however, it is difficult to adjust the parameters during the network training. Hang et al. [10] first performed pre-processing operations such as defogging and enhancement to fade the specific effects of foggy weather on the image and then used a complex image recovery network to recover and detect the image, but it requires separate training of the network model with pixel-level supervision. Sindagi et al. [11] proposed an image priori-based model for a foggy and rainy environment based on an unsupervised adversarial object detection algorithm. Although the above methods achieve significant results on synthetic datasets, they tend to overfit the provided training data with poor generalization, especially for real-world haze images.

For the domain transfer problem arising from artificially synthesized foggy images, most of the current domain adaptation algorithms are used to solve the domain transfer problem [12,13,14]. However, fog images with different concentrations have different characteristics, and existing studies tend to ignore this diversity. Previous domain adaptive methods only consider migration learning between the source and target domains while ignoring the joint optimization capability between image defogging, image enhancement, and object detection [15].

To overcome the shortcomings of the above object detection algorithm in a foggy environment, this paper proposes an object detection algorithm IDOD-YOLOV7 for pin autonomous vehicles under low-light foggy conditions, which focuses on the joint learning integration design of image defogging and image enhancement with object detection, to achieve the optimal performance of the whole detection system. The AOD (An All-in-One Network for Dehazing and Beyond) module compensates for the information loss during the convolution process by using parallel convolution layers to effectively improve the image defogging quality. To further improve the visualization of the defogged images, this paper introduces an adaptive image enhancement module (SAIP) with three hyperparameters (WB, Gamma, Contrast) that are learned adaptively by a lightweight CNN parameter predictor (CNN-PP). CNN-PP adaptively predicts hyperparameters based on the brightness, color, saturation, and color of the input image. After processing by the SAIP module, it can improve the external interference of low-light images due to light source transformations while recovering detailed texture information of the images. A joint optimization scheme is suggested to enhance the detection of low-light fog images by combining AOD, SAIP, and YOLOV7 [16] backbone detection networks. In addition, we use images with normal and different light intensities and different concentrations to train the proposed network. Taking advantage of the SAIP network, the IDOD-YOLOV7 method in this paper is able to detect foggy low-light images subjected to different concentrations. Figure 1b shows an example of the detection results of the proposed method in this paper.

The main contributions of this paper are as follows:

(1) We design a joint learning network for image enhancement and object detection (IDOD-YOLOV7), in which a parameter-tunable low-illumination image enhancement module (SAIP) is proposed, whose hyperparameters are predicted by a small network, to remove fog from the input image.
(2) We prepare a dataset for low-illumination fog object detection (FTOD) that is built by physical fog creation and artificial synthesis. This reduces the domain difference between the synthetic dataset and the real fog images on foggy days, avoids overfitting of the fog removal model, and improves the performance of object detection in real environments.
(3) We demonstrate the performance of our designed network architecture through various experiments. The experimental results show that our network outperforms existing methods on synthetic images both quantitatively and qualitatively.

The rest of the paper is organized as follows. In Section 2, the atmospheric scattering model of fog and the related properties of fog object detection are presented, providing a theoretical basis for the design of an object detection network architecture (IDOD-YOLOV7) in low-illumination foggy environments. Section 3 presents the detailed design and implementation of the IDOD-YOLOV7 architecture. Section 4 and Section 5 are the experimental sections and the conclusion section, respectively.

## 2. Related Work

In order to accurately describe the formation process of fog images, McCartney first proposed an atmospheric scattering model [17], where fog is produced when there are large amounts of water vapor and suspended tiny particles in the atmosphere under the absorption and scattering effects of natural light. The scattering effect of the particles causes light attenuation during transmission between the object and the sensor and adds a layer of atmospheric scattered light (Airlight) [18]. The detection system receives the light source imaging from two main parts; part of the light is reflected from the object to the detection system through particle attenuation, but from the light source (in this case light) through the scattering of atmospheric light formed by the particle reflection. The mathematical model of foggy sky imaging is obtained by this physical model as:

(1) $$I(x,\lambda )=e^{-\beta (\lambda )d(x)}R(x,\lambda )+L_{\infty }\left(1-e^{-\beta (\lambda )d(x)}\right)=D(x,\lambda )+A(x,\lambda )$$

where I(x,λ) is the foggy image acquired by the detection system; R(x,λ) is the fog-free image to be recovered; x is the position of the pixel in the image, λ is the wavelength of light; $L_{\infty }$ is the atmospheric light value at infinity; and $e^{-\beta (\lambda )d(x)}$ is the transfer function, the physical meaning of which is the proportion of light that can reach the detection system after particle attenuation. Most teams and scholars use the above atmospheric scattering model as the theoretical model for fog imaging when acquiring fog images through the detection system and performing image defogging. The main idea is to estimate the transfer function $e^{-\beta (\lambda )d(x)}$ or the atmospheric light $A\left(x,\lambda \right)$ from the foggy image based on various a priori knowledge or image processing tools and to recover the object image $R\left(x,\lambda \right)$ by substituting the solved parameters into the atmospheric scattering model [19].

For computational purposes, atmospheric transmittance is assumed to be $t\left(x\right)=e^{-\beta d\left(x\right)}$, the object reflected light to be $J\left(x\right)$, the object attenuated reflected light to be $D\left(x\right)=J\left(x\right)t\left(x\right)$, and the atmospheric scattered light value to be $A=L_{\infty }\left(1-t\right)=A_{\infty }\left(1-t\right)$, so as to obtain mathematical expression of the final atmospheric scattering model:

(2) $$I\left(x\right)=D+A=J\left(x\right)t\left(x\right)+A_{\infty }\left(1-t\left(x\right)\right)$$

By formulating a mathematical model, the imaging process of foggy images and the various elements contained in foggy images are explained. Although it is intuitive to perform image defogging based on a physical model, estimating atmospheric light values and transmittance are independent steps and errors can accumulate and may potentially amplify each other, resulting in poor image defogging quality.

(3) $$J\left(x\right)=\frac{1}{t\left(x\right)}I\left(x\right)-A\frac{1}{t\left(x\right)}+A$$

(4) $$J\left(x\right)=K\left(x\right)I\left(x\right)-K\left(x\right)+b$$

(5) $$K\left(x\right)=\frac{\frac{1}{t\left(x\right)}\left(I\left(x\right)-A\right)+\left(A-b\right)}{I\left(x\right)-1}$$

The two variables t(x) and A are integrated into a new variable K(x) represented by the transformation of Equations (3)–(5), where b is a constant deviation, which avoids cumulative errors in multiparameter estimation [20]. This provides the basis for the defogging model in the third section of this paper.

In order to reduce the impact of haze on image data and improve the accuracy of autonomous vehicle object detection, image dehaze is therefore an important component of late object detection. Image dehaze algorithms are currently divided into four categories: the first category is based on image enhancement dehaze methods, usually using traditional image processing methods to improve image contrast and enhance image brightness, gradient, and other feature information, which does not defog from the formation mechanism of fog, resulting in defogged images prone to color deviation, loss of detail and other phenomena. The second category is the physical model-based approach, which establishes a degraded physical model of fog based on the scattering mechanism of atmospheric light and uses image-priori methods such as saturation prior [21], sparsity prior, and regular probability prior for dehaze [22]. For example, He et al. [17] used a dark channel priori assuming that the presence of the color channel has low values under the background region of the object in a clear image, and used an atmospheric scattering model based on this priori knowledge for the dehaze. Zhu et al. [23] proposed a color decay priori algorithm for the estimation of fog-free image projection and recovery based on the fact that the greater the fog concentration, the greater the depth of field. The above algorithms have low time complexity, but it is more difficult to find general prior knowledge in complex traffic environments, leading to an inaccurate estimation of the transmission map and an incomplete dehaze and color distortion. The third category is based on neural networks and deep learning methods, which are divided into two types; one is a network architecture based on atmospheric scattering models such as the AOD-Net network proposed by Li et al., and the Dehazenet network proposed by Cai et al., based on CNN [24,25]. The network architecture relies excessively on atmospheric scattering models. Another end-to-end deep learning approach for direct dehaze, such as Liu et al. [26], proposes a dehaze network with a grid-like shape, which effectively alleviates the bottleneck problem in traditional multi-scale fusion dehaze methods through an attention mechanism. Yang et al. [27] proposed a feature fusion-based image-dehazing network, which defogs by learning content and style features while maintaining the color characteristics of the original image. Dong et al. [10] used the U-Net architecture to correct the missing spatial information in high-resolution features using back-projection techniques and non-neighboring features. Chen et al. [28] proposed a codec network based on the contextual information gated fusion, but lacked information interaction between features in the encoding and decoding stages. Yang et al. [29] proposed a codec network with a multi-scale feature aggregation network, but the information of different scale features was not retained during the successive downsampling in the coding stage of the network, resulting in the loss of image features and affecting the recovery of high frequency details of images. The above end-to-end network cannot fully utilize the multi-scale feature information of the image, resulting in incomplete image dehaze. In addition, only pixel-by-pixel recovery is considered during the training process, which results in some color distortion in the defogged image despite the high peak signal-to-noise ratio index.

The ability to sense complex traffic environments is crucial for autonomous driving; however, current detection models trained under normal weather conditions in harsh natural environments such as fog are usually not adequate for object detection in adverse weather conditions because of the large amount of fog noise in the images captured by the camera. There are currently four main approaches to object detection in harsh environments, the first of which is based on image pre-processing, such as image denoising and image enhancement to remove the specific effects of fog on images [30]. However, as both fog noise and image texture are high-frequency small-scale information, model training by image pre-processing can easily lead to the loss of image detail information. The second is a priori-based approach for image dehaze and object detection, such as the priori-based domain adaptive object detection under rain and fog conditions proposed by Sindagi et al. [11] The third one uses a two-branch joint learning network [9], where one network is used for image dehaze operations and the other for object detection. Since the two network branches share the feature extraction layer, it is difficult to balance the two tasks during training. The fourth one uses end-to-end adaptive learning to align detection targets in clear and fogged images for object detection. Liu et al. [31] proposed the IA-YOLO network for object detection in complex environments, where object labels and bounding box coordinates are predicted by a single CNN. This method leads to a high rate of missed detection due to incomplete defogging. However, for the current low illumination, low contrast, and small object environment, the current detection model in harsh environment detection also has some application value. The SVA-SSD algorithm proposed by Shahin et al. [32] has significant results in object detection in a low contrast environment. A lightweight multi-scale small object detection algorithm was proposed by Li et al. [33]. The algorithm effectively improves small object detection accuracy while reducing the model complexity, but the detection performance is degraded for bad weather conditions. In this paper, we mainly use a joint optimization based on an image dehaze module (AOD), image enhancement module in (SAIP), and single-stage detector YOLOV7 module for autonomous vehicle object detection in a low-light foggy traffic environment [16], which improves its accuracy and real-time object detection in foggy conditions.

## 3. Material and Methods

The reduced visibility of images due to low-light and foggy conditions seriously affects the accuracy of object detection for autonomous vehicles and poses a great challenge to the vehicle’s environmental perception capability. In order to better solve this practical problem, this paper creates a low-illumination foggy weather traffic object detection dataset (FTOD) and proposes an object detection algorithm IDOD-YOLOV7 for autonomous vehicles under foggy weather conditions, which reveals more potential information inside the image by eliminating foggy interference. The whole network framework consists of an AOD module, an adaptive image enhancement module (SAIP), and a YOLOV7 detection module. The AOD module first estimates the two parameters of the atmospheric scattering model to obtain a preliminary defogged image. Then, the model resizes the image to 256 × 256 and feeds it to the SAIP module for parameter optimization to further improve the image dehaze quality and enhance the image for object detection in foggy weather in a weakly supervised manner. Finally, the processed image from the SAIP module is used as the input to the YOLOV7 detector and used for traffic environment object detection.

### 3.1. Foggy Object Detection Dataset

As it is very difficult to obtain paired foggy/non-foggy images of the traffic road environment in the same scene, this paper generates foggy traffic images by physical fog creation and artificial synthesis. First, two models (SONY) FDR-AX700 4K HDR digital cameras were used simultaneously, one for clear image acquisition and the other for simulating fog scenes of different concentrations by adding different concentrations of fogging flakes in front of the camera lens, while the environment was fogged by artificial spray in order to further enhance the realism of the fog scenes. A foggy traffic object detection dataset, FTOD, was created, containing 5100 pairs (1000 pairs of physical fogging, 4100 pairs of VOC_foggy synthesis) of fog-free and fogged images.

The VOC_foggy dataset is a synthetic dataset built on the basis of the classical VOC dataset based on the atmospheric scattering model. In order to form traffic images in low-illumination foggy environments, this paper chooses data containing five categories to add fog. To avoid the computational overhead of generating fog images during the training process, the VOC_foggy dataset was constructed offline. According to Equations (1) and (2), the fog image I(x) is obtained by the following steps, where d(x) is 0.4 times the Euclidean distance from the current pixel to the center pixel, and multiple different levels of fog can be added to each image by setting A=0.5 and β=0.01×i+0.05, where i is an integer from 0 to 9.

**Figure 2:**
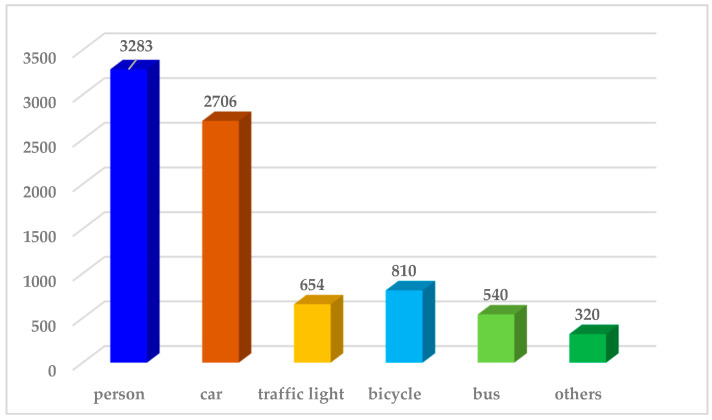
The label distributions in the proposed FTOD. Most of the annotated objects are cars (2706 labeled), pedestrians (3283 labeled), and bicycle (810 labeled).

In order to determine the effect of fog on detection accuracy at different concentrations, this paper divides the fogged images into three difficulty classes: heavy, medium, and light, according to the fog coverage ratio (FCR) of the detected objects. By combining FCR and human eye visual effects, image numbers 1~2600 are classified as easy level, 2601~3600 as normal level, 3601~4600 as difficult level, and 4601~5100 as particularly difficult level. Figure 2 shows the label distribution of the FTOD dataset.

### 3.2. Image Dehazing Model

The AOD-NET (end-to-end defogging network) is optimized based on the atmospheric scattering model, which is direct image recovery by a lightweight CNN network and is therefore highly portable. In this paper, based on this feature of AOD-NET, we optimize the original network architecture and construct an end-to-end smart driving vehicle object detection algorithm under low-illumination fog conditions by fusing its network characteristics with the YOLOV7 detection network.

**Figure 3:**
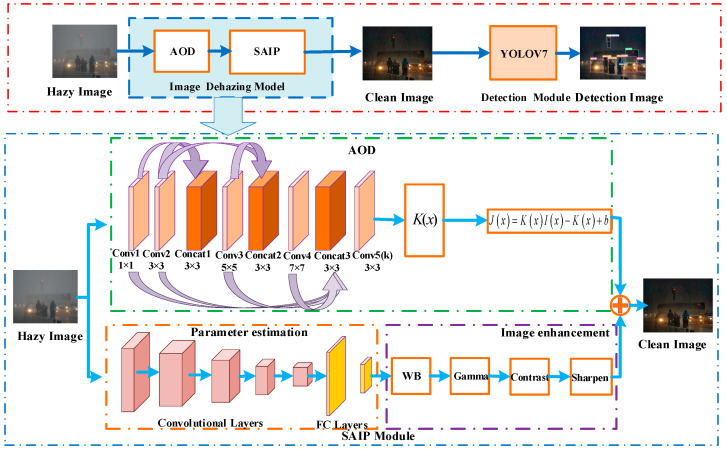
IDOD-YOLOV7 network structure diagram. AOD for image defogging module, SAIP for image enhancement module, and YOLOV7 for object detection module.

The original AOD-NET network consists of two modules, divided into an estimation module and an image recovery module. Since the atmospheric scattering model-based image defogging algorithm produces error accumulation in the process of parameter estimation, it results in incomplete image defogging image distortion. Therefore, in this paper, a self-adaption image processing module (SAIP) is added following the AOD image defogging module. This module includes four image enhancement weights (pixel-level filters), including white balance (WB), gamma correction, contrast enhancement, and image sharpening [34]. WB can eliminate the chromatic aberration caused by atmospheric light, the gamma correction can restore the detail information in darker areas, the contrast enhancement can improve the global visibility of thick fog areas, and the image sharpening can effectively improve the visualization of the defogged image. The structure of the Image Dehazing Model is shown in Figure 3.

#### 3.2.1. AOD Module

The AOD Module is used to perform image defogging based on the atmospheric scattering model. This module adopts a joint learning method to unify A and t(x) for learning, which can effectively improve the accuracy of parameter estimation. The module has two parts, as shown in Figure 3: the parameter estimation module and the image recovery module, where the parameter estimation module uses a 5-layer convolution to estimate K(x) for the input image $I_{x}$, and the image recovery module uses the estimated K(x) value and b output to recover the image.

The K(x) estimation module mainly uses five convolutional layers to fuse multi-scale information, and in order to link coarse-scale features with fine-scale features effectively, the parallel convolutional layer “Concat” is used for the connection. As shown in Figure 3, Concat1 connects Conv1 and Conv2, Concat2 connects Conv2 and Conv3, and Concat3 connects Conv1, Conv2, Conv3 and Conv4. Using different parallel convolutional layers can effectively compensate for the information loss during the convolution process. The image recovery module generates the recovered image based on the parameter estimation gate module obtained from K(x). The recovered image is generated by the element summation layer and element multiplication layer according to Equation (5).

#### 3.2.2. SAIP Module

Generally, the filter parameters are manually adjusted based on experience for image correction and image enhancement operations. This not only leads to poor image enhancement due to large parameter errors, but also makes the system less adaptive. To effectively address this issue, this paper uses small CNN networks to estimate the four filter parameters of the SAIP module.

The SAIP module consists of a pixel-level filter and a sharpening filter. The pixel-level filter consists of three adjustable parameters: white balance, gamma correction, and contrast enhancement, which are mainly used to smooth the image after defogging to improve the visualization of the image. The mapping functions of the pixel-level filters map the input image $P_{i}$ to the three color channels (R, G, B) of the output image $P_{o}$, respectively. Table 1 shows the mapping functions of the three filters and the parameters to be optimized. The contrast filter sets a linear interpolation between the original image and the fully enhanced image by the input parameters. The definition of $En(P_{i})$ in the mapping function is as follows:

(6) $$L\left(P_{i}\right)=0.27r_{i}+0.67g_{i}+0.06b_{i}$$

(7) $$EnL\left(P_{i}\right)=\frac{1}{2}\left(1-\cos\left(\pi \times \left(L\left(P_{i}\right)\right)\right)\right)$$

(8) $$En\left(P_{i}\right)=P_{i}\times \frac{EnL\left(P_{i}\right)}{L\left(P_{i}\right)}$$

where $P_{i}$ denotes the input pixel value, $r_{i},g_{i},b_{i}$ denote the image RGB color channel pixel values, respectively, and the $P_{o}$ denotes the mapped output pixel value.

The image sharpening filter mainly uses image sharpening to compensate for contours and highlight edge information in order to make the image clearer after defogging, and the process of image sharpening can be described as follows [35].

(9) $$F\left(x,\lambda \right)=I\left(x\right)+\lambda (I\left(x\right)-Gau\left(I\left(x\right)\right)$$

where I(x) is the input image, $Gau\left(I\left(x\right)\right)$ is the Gaussian filter, and λ is the positive scale factor.

The SAIP module is based on the CNN network for parameter optimization. Since CNN extracts high-resolution image feature information, it leads to a large amount of wasted computer resources. Therefore, the acquired high-resolution fogged images are adjusted to low-resolution images (256×256) and the image filtering parameters are extracted. The above filters are applied to the low-resolution defogging images for image enhancement after defogging by the AOD module.

The fogged images are downsampled by a small CNN network, which not only speeds up the parameter estimation but also reduces the number of model computational parameters [36]. As shown in Figure 3, the defogged images are first reduced to 256×256 by bilinear differences before parameter estimation, and then hyperparameter estimation is performed by five convolution blocks and two fully connected layers, where each convolution block consists of 3×3 convolution layers, and ReLu activation functions with output channels of 16, 16, 32, 32, and 32, respectively. For white balance (WB), gamma correction, contrast enhancement, and image sharpening filters, the number of parameters to be estimated is small, and in order to reduce the waste of computational resources and improve network efficiency, a small CNN network is used to downsample the defogged images. This not only speeds up the parameter estimation but also reduces the number of computational parameters of the model. Before parameter estimation, the defogged images are bilinearly differenced to reduce the resolution to 416 × 416, and the hyperparameter estimation is relatively reasonable and efficient for low-resolution images with five convolutional blocks and two fully connected layers.

### 3.3. Detection Network Module

In order to improve the accuracy and real-time object detection for autonomous driving in low-light and foggy weather conditions, the latest version of YOLOV7 is selected as the detection network. YOLO series is a fast object detection algorithm, whose main characteristics are being fast and lightweight based on certain detection accuracy [37]. Therefore, the selected YOLO detection algorithm is well suited for object detection of autonomous vehicles when deployed in low-light fog environments.

YOLO has continuously improved its detection accuracy and speed from V1-V7. YOLOV7 differs significantly from previous versions in terms of model structure (CSP->ELAN), partial convolution strategy approach (Conv->RepConv), and label assignment approach (IOU, simota->Coarse to fine deep supervision approach) [38]. YOLOV7 focuses on the training process of optimization to make the training process more costly and thus improve accuracy without increasing computational parameter consumption. It can reduce the number of parameters by 40% and the computational effort by 50% compared to Sota’s object detection method. Combining the above features, the same network architecture and lost function of YOLOV7 are used for the object detection module in low-light foggy environments. The algorithm is used for object detection after defogging images to achieve road environment awareness for autonomous driving in foggy and bad weather conditions, optimize control decisions, and improve the safety of autonomous vehicles in bad weather conditions [39].

**Figure 4:**
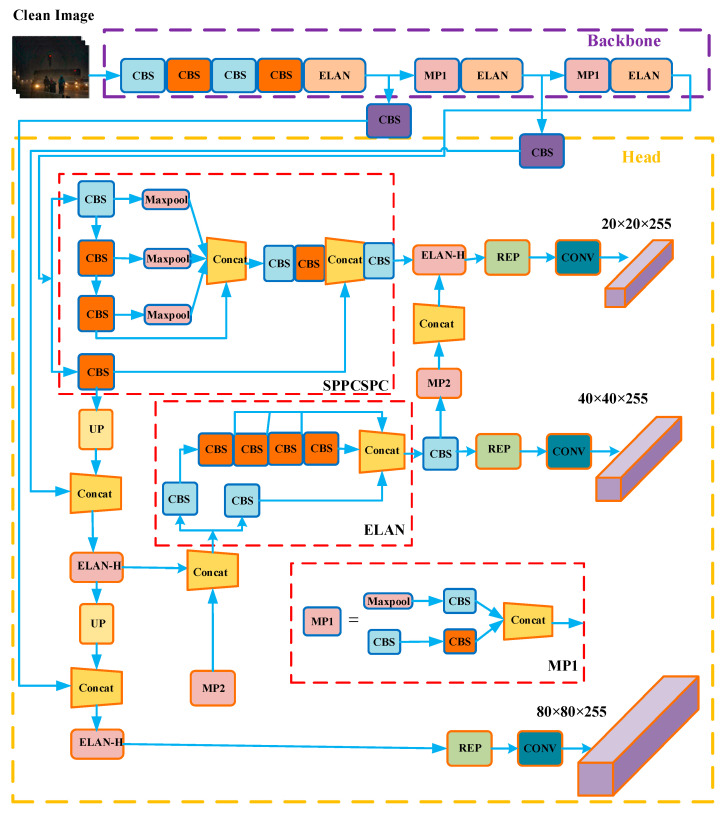
The network architecture diagram of Yolov7 contains four general modules: input terminal, backbone, head, and prediction, and five basic components: CBS, MP, ELAN, ELAN-H, and SPPCSPC.

The architecture of YOLOV7 is shown in Figure 4, in which an extended ELAN (E-ELAN) based on ELAN is proposed in the network architecture. Without destroying the original gradient path, the learning capability of the network is continuously enhanced by using methods such as extension and merging bases. Group convolution is used to extend the channels and bases in the computation process. The YOLOV7 network consists of three parts: input, backbone, and head. When the backbone is used to extract features, the entire backbone layer consists of several BConv layers, E-ELAN layers, and MPConv layers alternately halving the aspect, doubling the channels, and extracting features. Head is used for prediction, consisting of SPPCPC layers, several BConv layers, several MPConv layers, several Concat layers, and a RepVGG block layer that subsequently outputs three Heads. After the Head outputs three feature maps, it outputs three unprocessed predictions of different sizes through the three REP and Conv layers, respectively.

### 3.4. Multi-Source HybrIDOD Weather Data Training

A multi-source hybrid weather data training scheme is adopted for IDOD-YOLOV7 to improve the robustness of the system. The 1/4 rainy weather image and low-light image are selected in the network training process. In the presence of fog training data and other weather training data, the whole network is trained end-to-end with the loss of YOLOV7, which ensures mutual adaptation between the modules in IDOD-YOLOV7. The multi-source hybrid weather data training mode ensures that IDOD-YOLOV7 is capable of adapting the images according to the content of each image, resulting in high detection performance. In the case of mixed dataset training data, the entire pipeline is trained end-to-end with YOLOV7 detecting loss, ensuring mutual adaptation among the modules in IDOD-YOLOV7. The mixed data training mode ensures that IDOD-YOLOV7 is able to perform adaptive processing of images based on the content of each image, resulting in high detection performance. Meanwhile, the use of a real data set (FTOD) can effectively solve the domain transfer problem due to synthetic data, making the training data more relevant to the real low-illumination foggy traffic environment, thus improving the generalization ability and robustness of the plus detection network.

## 4. Experiments and Results

This section systematically analyzes and evaluates the detection module and the dehaze module of the algorithm in a real low-light foggy environment with different concentrations. The experimental results of the algorithm proposed in this paper under a foggy traffic environment are summarized. In order to verify the detection performance of the IDOD-YOLOV7 network structure in a low-light foggy environment, first, the defogging module is compared with the existing classical defogging methods DCP [18], AOD-NET [20], MSCNN [40], GFN [41], GCANET [29], FFA-NET [42], DCPDN [43], EPDN [44], and Dehazenet [25]. Then, it is compared with the existing detection methods Fast R-CNN, Faster R-CNN, SSD, RetinaNet, and YOLOV3 methods [45]. Finally, the detection algorithm is compared with YOLOV7 [16] and various defogging algorithms such as AOD-NET, CAP [46], MSCNN, FFA-NET, and Dehazenet for a comprehensive comparison. The above experiments are conducted on a PC equipped with NVIDIA GeForce GTX 2080Ti GPU.

### 4.1. Implementation Details

The backbone network for all experiments in the IDOD-YOLOV7 method is New ELANCSP. Data enhancement methods such as image flipping, cropping, and transformations are used during the training process to extend the training dataset. In addition, a random approach is used to resize the image to (64n×64n) where n∈[9,19]. IDOD-YOLOV7 is trained with Adam optimizer, using SyncBatchNorm multi-GPU for distributed training, DDP parameters are set to default values, the maximum number of workers is eight, there are 200 Epochs, initial learning rate $10^{-4}$, and the batch size is four. IDOD-YOLOV7 predicts the position of detection frames by different scales. There are three anchors on each scale. Three different image input scales are set with multi-scale training enabled, and one scale is randomly selected at certain iterations during training to enhance the robustness of the model. Experiments are performed using Pytorch and run on GPU.

For the IDOD-YOLOV7 network structure, this paper uses a joint training approach to train the defogging sub-network and the detection network separately. First, the detection network YOLOV7 is not trained from scratch using pre-trained weights trained on the MS COCO dataset [47], but is trained by migration learning in combination with the dataset presented in this paper. Then, the SAIP module is reproduced by sharing the first five convolutional layers of YOLOV7 and the network is trained jointly with mixed data.

The loss function consists of three parts, which are divided into three parts: Regression Loss, Object Confidence Loss, and Classification Loss. Among them, the object confidence loss and classification loss use BCE With Logits Loss (cross-entropy loss), and the regression loss uses CIoU loss [48]. The total loss function is the sum of the above three losses, as follows.

(10) $$L_{DCF-YOLOV7}\left(t_{p},t_{\mathrm{gt}}\right)=\sum_{k=0}^{K}\left[\alpha_{k}^{\mathrm{balance}\;}\alpha_{\mathrm{box}}\sum_{i=0}^{S^{2}}\sum_{j=0}^{B}I_{kij}^{\mathrm{obj}}L_{\mathrm{CloU}}+\alpha_{\mathrm{obj}}\sum_{i=0}^{S^{2}}\sum_{j=0}^{B}I_{kij}^{\mathrm{obj}}L_{\mathrm{obj}}+\alpha_{\mathrm{cls}}\sum_{i=0}^{S^{2}}\sum_{j=0}^{B}I_{kij}^{\mathrm{obj}}L_{\mathrm{cls}}\right]$$

where K is the output feature map, $S^{2}$ is the number of cells, B is the number of anchors on the cells, and α is the weight value of the corresponding term. In this paper, $\alpha_{\mathrm{box}}=0.05,\alpha_{\mathrm{cls}}=0.3,\alpha_{\mathrm{obj}}=0.7$, $I_{kij}^{\mathrm{obj}}$ are the kth feature map, $t_{\mathrm{gt}}$ and $t_{p}$ are the ground-truth vector and the prediction vector, respectively. $\alpha_{k}^{\mathrm{balance}\;}$ is the weight of the output feature map at each scale, and the default values are [4.0,1.0,0.4], which correspond to 80×80,40×40,20×20 feature maps in turn. Where the three weight values are based on the default values of the detection network, usually the confidence loss takes the maximum weight and the rectangular box loss and the classification loss take the second weight.

Bbox Regression Loss:

(11) $$L_{CIoU}=1-IOU+\frac{\rho^{2}\left(\;b,b^{gt}\right)}{C^{2}}+\frac{\rho^{2}\left(w,w^{gt}\right)}{C_{w}^{2}}+\frac{\rho^{2}\left(h,h^{gt}\right)}{C_{h}^{2}}$$

(12) $$\rho (\cdot )={\left\|b-b^{gt}\right\|}_{2}$$

where b is the prediction box, $b^{gt}$ label box; $w^{gt}$ and $h^{gt}$, are the width and height of the label box, w and h are the width and height of the prediction box, ρ is the distance between the center points of the two boxes, and C is the farthest distance between the boundaries of the two boxes.

Object Confidence Loss:

(13) $$L_{\mathrm{obj}\;}\left(p_{o},p_{\mathrm{iou}\;}\right)={\mathrm{BCE}}_{\mathrm{obj}}^{\mathrm{sig}}\left(p_{o},p_{\mathrm{iou}\;};w_{\mathrm{obj}\;}\right)$$

where $BCE_{cls}^{sig}$ denotes binary cross-entropy loss, $w_{obj}$ denotes positive sample weights, $P_{o}$ denotes the object confidence score in the prediction frame, and $P_{iou}$ the prediction frame and the iou value of the corresponding object frame, which is used as ground-truth. Both calculate the binary cross-entropy to obtain the final object confidence loss.

Classification Loss:

(14) $$L_{\mathrm{cls}}\left(c_{p},c_{\mathrm{gt}}\right)={\mathrm{BCE}}_{\mathrm{cls}}^{\mathrm{sig}}\left(c_{p},c_{\mathrm{gt}};w_{\mathrm{cls}}\right)$$

where $BCE_{cls}^{sig}$ denotes binary cross-entropy loss, $w_{cls}$ denotes positive sample weights, and $c_{p}$ and $c_{gt}$ denote the predicted and true values of the corresponding categories, respectively. The two calculate the binary cross-entropy to get the final category loss.

Initialization:

In this paper, a random Gaussian distribution (mean μ = 0, standard deviation σ = 0.001) is used for initialization, and the learning rate decreases by half from 0.00001 to 3.125 × 10^−4^ per 100,000 iterations. In the detection sub-network, the trained YOLO model on the MS COCO dataset is used, and the fully trained weights of this model are fine-tuned instead of randomly initializing the weights.

To accurately measure the performance of the IDOD-YOLOV object detector, the *AP* metrics of the accuracy and completeness curves (PRC) were used, where the precision ($P_{r}$) and recall ($R_{e}$) for different thresholds are expressed as follows:

(15) $$P_{r}=\frac{TP}{TP+FP}$$

(16) $$R_{e}=\frac{TP}{TP+FN}$$

where TP, FP, and FN are the number of true positives, false positives, and false negatives, respectively. The AP defined as follows:

(17) $$AP=\int_{0}^{1}P_{r}\left(R_{e}\right)d_{R_{e}}$$

The average accuracy (mAP) of the object detector is expressed in terms of the average AP value of all object classes and is calculated as follows:

(18) $$mAP=\frac{1}{M}\overset{M}{\underset{z=1}{\sum }}AP_{z}$$

where M is the number of object categories.

### 4.2. Performance Evaluation of Image Dehazeing Model

For object detection in low-light foggy environments, the quality of image dehaze in the preliminary stage affects the accuracy of object detection in the next stage. In this paper, we compare the image dehaze module (AOD + SAIP) with defogging methods based on a priori DCP and deep convolutional neural networks (AOD-NET, MSCNN, GFN, EPDN, Dehazenet, FFANet, PSD [49], CycleGan [50], YOLY [51]). For an accurate comparison, retraining on the same training dataset and evaluation on the same test dataset (SOTS [52], HAZE [53], FTOD) were used. The objective evaluation metrics PSNR (Peak Signal to Noise Ratio), SSIM (Structural similarity) [54], and MSE (Mean Square Error) were used to evaluate the effectiveness of the above defogging algorithms.

PSNR (Peak Signal to Noise Ratio) a full reference image quality evaluation index. Given a clear image I of size m×n and a noisy image K, the mean square error MSE is:

(19) $$\mathrm{MSE}=\frac{1}{mn}\overset{m-1}{\underset{i=0}{\sum }}\overset{n-1}{\underset{j=0}{\sum }}{\left[I(i,j)-K(i,j)\right]}^{2}$$

Then, PSNR (dB) is defined as:

(20) $$\mathrm{PSNR}=10\cdot \log_{10}\left(\frac{MAX_{I}^{2}}{MSE}\right)$$

where $MAX_{I}^{2}$ is the maximum possible pixel value of the image.

SSIM (structural similarity), which is also a full-reference image quality evaluation index, measures image similarity in terms of brightness, contrast, and structure, respectively.

(21) $$\mathrm{SSIM}(x,y)=\frac{\left(2\mu_{x}\mu_{y}+c_{1}\right)\left(2\sigma_{xy}+c_{2}\right)}{\left(\mu_{x}^{2}+\mu_{y}^{2}+c_{1}\right)\left(\sigma_{x}^{2}+\sigma_{y}^{2}+c_{2}\right)}$$

where $\mu_{x}$, $\mu_{y}$ denote the mean values of images x and y, respectively, $\sigma_{x}$, $\sigma_{y}$ denote the variance of images x and y, respectively, and $\sigma_{xy}$ denotes the covariance of images x and y; $c_{1}$, $c_{2}$, $c_{3}$ are constants. In order to avoid the case that the denominator is 0, we usually take $c_{1}={\left(k_{1}\times l\right)}^{2}$, $c_{2}={\left(k_{2}\times l\right)}^{2}$, $c_{3}=0.5c_{2}$, generally $k_{1}$ = 0.01, $k_{2}$ = 0.03, l = 255. Then, SSIM takes the value range [0, 1].

**Figure 5:**
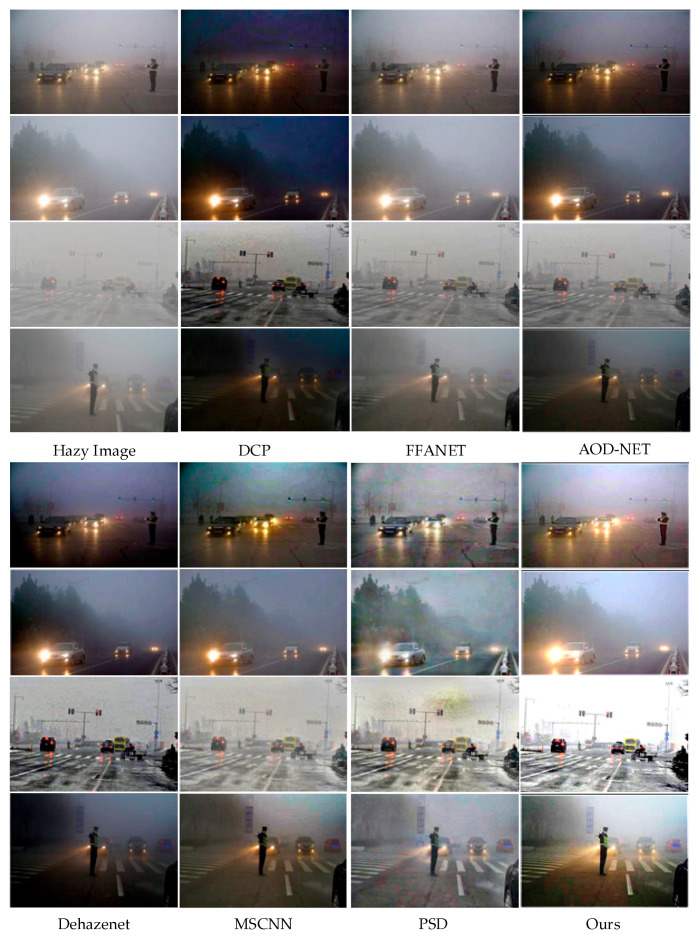
Qualitative comparison of different methods on low-light foggy traffic images.

The results of the objective evaluation of the defogged images for different datasets are shown in Table 2. It can be seen that the defogging method using DCP performs poorly. FFA-NET demonstrates strong fitting ability on the SOTS test set, but loses its advantage when the test sample is not consistent with the training data. In contrast, the proposed (AOD + SAIP) method outperforms the above algorithms in terms of objective evaluation metrics PSNR, SSIM, CIEDE, and image dehaze efficiency. Figure 5 shows the effect of using the above defogging methods. DCP algorithm has obvious color distortion in the defogged image of scenes with a large fog concentration. The defogging method based on the atmospheric scattering model is less effective due to the inability to accurately estimate the atmospheric light values. End-to-end-based direct image defogging is superior to other methods based on indirect estimation of atmospheric scattering model, but produces excessive defogging. Other deep learning-based methods have different degrees of image artifacts and incomplete defogging. The defog method in this paper not only removes most of the haze from the image but also improves the contrast and saturation of the image, thus effectively improving the image quality and providing a basis for the next step of object detection.

**Figure 6:**
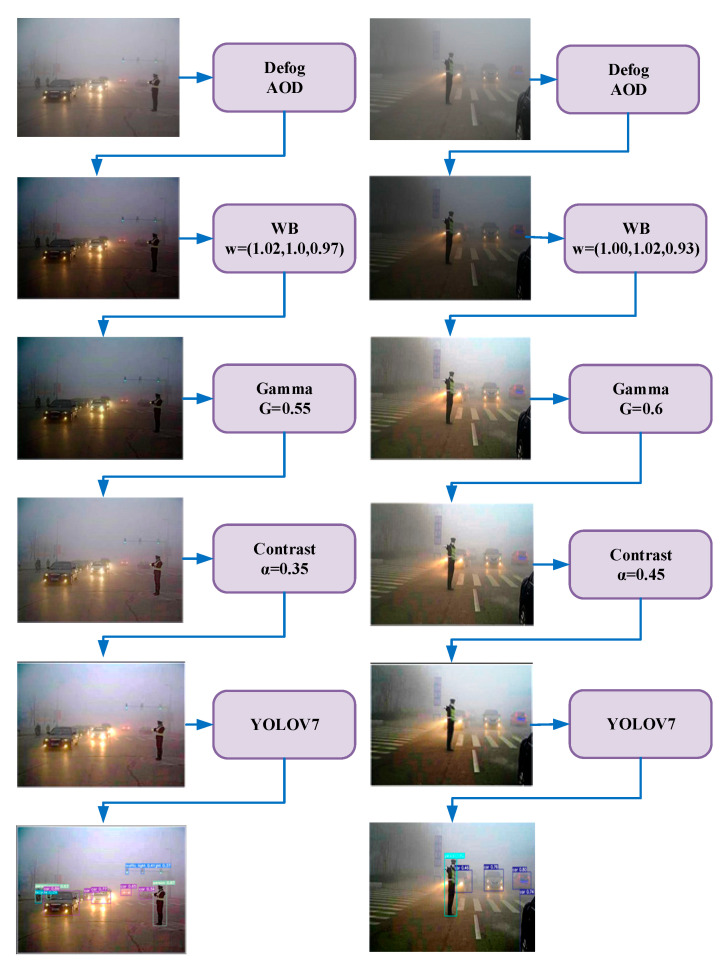
Examples of learned SAIP modules and their filtering outputs.

Two examples of how the SAIP module image enhancement parameters (WB, Gamma, Contrast) are predicted by a CNN-PP network are given in Figure 6. The CNN-PP network is able to learn a set of parameters for each image based on specific information about the brightness, color, hue, and fog concentration of each low-illumination fog image. To give a more visual representation of the processing effect of each filter, the parameter values of each filter and the processed image are shown in Figure 6. The defogged images are processed by the SAIP module to improve the visualization of low-illumination images and improve the detail information of the images, which is beneficial to the subsequent object detection.

### 4.3. Object Detection Results

To validate the performance of the joint optimization model IDOD-YOLOV7 proposed in this paper for object detection in low-light foggy environments, cross-sectional and longitudinal comparison experiments are conducted on the same test dataset. First, the IDOD-YOLOV7 algorithm is compared with the current state-of-the-art CNN object detectors Fast R-CNN, Faster R-CNN, SSD, RetinaNet, and YOLOV3 methods. Table 3 shows the detection results of different detectors at different concentrations of fog images. As can be seen from the table, the object detection accuracy using IDOD-YOLOV7 algorithm is better than the above detection algorithms in a low-light fog environment.

**Figure 7:**
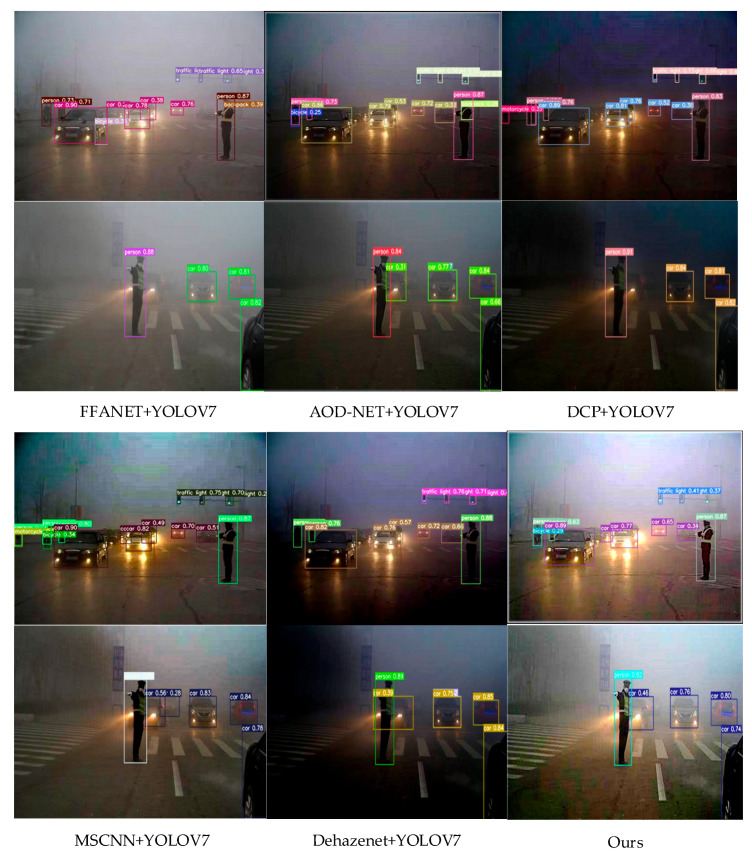
Object detection results on natural low-light foggy traffic images with a confidence thresh-old of 0.7, displayed on the dehazed results.

To verify the effect of the defogging module (SAIP) on the later object detection algorithm, comparison experiments of AOD-NET + YOLOV7, DCP + YOLOV7, MSCNN + YOLOV7, and Dehazenet + YOLOV7 for foggy object detection were conducted. Figure 7 shows the detection effect plots for different combinations mentioned above. From the figure, it can be seen that the accuracy and the leakage rate of fog concentration detection using IDOD-YOLOV7 algorithm are significantly better than other algorithms in moderate and heavy fog. The first three rows of Table 4 compare the mean accuracy (mAP) of the above methods on the three different test sets. As can be seen from the table, the detector performance in fog object detection is greatly improved by using this defogging + detection mode. Among them, MSCNN + YOOV7 improves the detection efficiency of fog concentration by 8%, and both AOD-NET + YOLOV7 and Dehazenet + YOLOV7 improve, but perform slightly worse compared to MSCNN + YOOV7. The joint optimization model proposed in this paper outperforms the above combined algorithms in terms of average accuracy for all haze conditions (light, medium, or heavy).

In this paper, a mixed training set with different concentrations is randomly generated in the SONY dataset. The last three rows of Table 4 show the results of retraining the tuned IDOD-YOLOV7 on this training set. Although the results of the mixed dataset training are slightly inferior to the average detection accuracy specifically applied to specific fog concentrations, they perform well in terms of average accuracy for all fog conditions (light, medium, or heavy).

The method proposed in this paper has certain superiority in low-light fog traffic environments. Since it is difficult to obtain fog and non-fog images of the same scene, the current advanced fog detection algorithms use conventional datasets (synthetic datasets) for model training, so there is a serious domain transfer problem and their detection performance is far inferior to the method proposed in this paper in real low-light fog traffic environments.

### 4.4. Ablation Study

**Figure 8:**
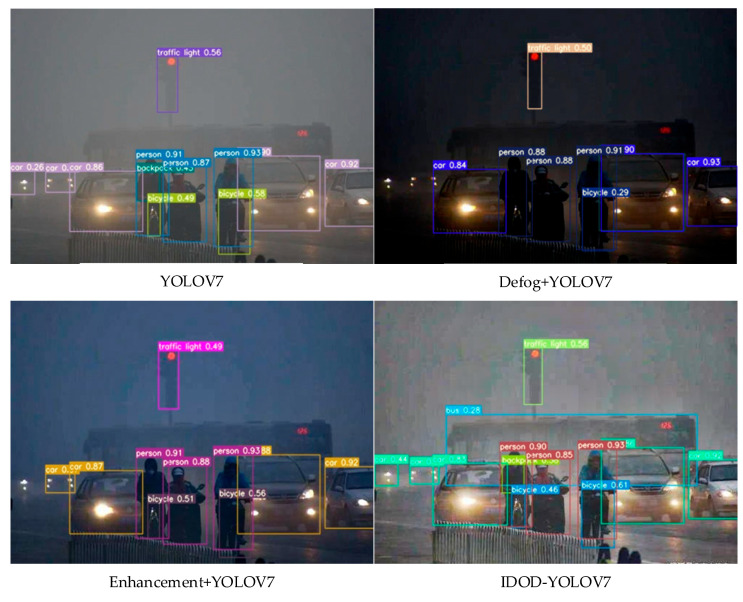
Ablation study object detection results of YOLOV7, Defog + YOLOV7, Enhancement + YOLOV7, and our IDOD-YOLOV7 on low-light foggy traffic images. The proposed method has better detection performance with fewer missed and wrong detections.

In this subsection, ablation experiments are performed using different settings in order to verify the effectiveness of the image enhancement module (image denoising + image filtering) proposed in this paper for late object detection. The IDOD-YOLOV7 method is compared with the benchmark YOLOV7, Enhancement + YOLOV7, and Defog + YOLOV7 on the same three test datasets. Table 5 shows the mAP of the test datasets using the three fog concentrations. Detection performance is improved using Enhancement + YOLOV7 and Defog + YOLOV7 over YOLOV7 alone in a low-light foggy environment. Figure 8 shows the comparison of object detection visualization results in a dense fog scene. Compared with the generated image enhancement model (enhancement + YOLOV7) and the generated image defogging model (Defog + YOLOV 7), the IDOD-YOLOV algorithm not only effectively improves the visibility of the low-light fog images, but also improves the accuracy of object detection and reduces the rate of missed detection. The detection accuracy (mAP) is improved by 24% compared to enhancement + YOLOV7 in heavy fog situations and by 26% compared to Defog + YOLOV7. The ablation study verifies that joint learning image defogging and image enhancement is effective for object detection algorithms.

### 4.5. Model Efficiency Analysis

The proposed method in this paper outperforms other detection methods in terms of object detection accuracy, but in order to effectively improve the detection accuracy, image enhancement and image defogging are required for low-illumination fog images, so the computational load is greater than that of a single object detection algorithm. The time complexity, space complexity, and detection speed of the detection method in this paper compared with other methods are shown in Table 6. The time complexity, i.e., the number of operations of the model, is evaluated using Floating-point Operations (FLOPs) for the time complexity of a CNN model. The time complexity of a single convolutional layer is represented by Equation (22):

(22) $$Time\sim O(M^{2}\cdot K^{2}\cdot C_{in}\cdot C_{out})$$

where M denotes the edge length of the output feature map for each convolution kernel, K the edge length for each convolution kernel, and $C_{in}$ the number of channels for each convolution kernel, and $C_{out}$ denotes the number of convolution kernels that the convolution layer has, i.e., the number of output channels. Among them, the output feature map size itself is determined by four parameters: input matrix size X, convolution kernel size K, Padding, and Stride, which are expressed as follows.

(23) M=(X−K+2×Padding )/ Stride +1

The overall time complexity of the convolutional neural network is expressed as follows:

(24) $$\;\mathrm{Time}\;\sim O\left(\sum_{l=1}^{D}M_{l}^{2}\cdot K_{l}^{2}\cdot C_{l-1}\cdot C_{l}\right)$$

where D denotes the network depth, l denotes the lth convolutional layer of the neural network, and the number of output channels of the lth convolutional layer of the $C_{l}$ neural network $C_{out}$.

The spatial complexity consists of two parts: the total number of parameters and the output feature map of each layer. The number of parameters is the total number of weight parameters for all layers of the model with parameters (i.e., the model volume, the first summation expression below); the feature map is the size of the output feature map calculated for each layer of the model during the real-time run (the second summation expression below).

(25) $$\;\mathrm{Space}\;\sim O\left(\sum_{l=1}^{D}K_{l}^{2}\cdot C_{l-1}\cdot C_{l}+\sum_{l=1}^{D}M^{2}\cdot C_{l}\right)$$

where K denotes the size of the convolution kernel, C denotes the number of channels, and D denotes the number of layers.

The time complexity determines the training/prediction time of the model and the spatial complexity determines the number of parameters of the model. From Table 6, it can be seen that the IDOD-YOLOV7 network architecture in this paper increases the time complexity and spatial complexity of the model due to the introduction of SAIP and AOD modules, which increases by 4% and 26%, respectively, compared to the baseline YOLOV7, but the detection speed increases by 10.7%.

## 5. Discussion and Conclusions

In this paper, we propose an object detection algorithm IDOD-YOLOV7 for low-light foggy traffic environments by joint learning of image defogging and image enhancement. The IDOD-YOLOV7 algorithm is compared with various advanced dehaze and object detection algorithms. The image defogging module (AOD) and image enhancement module (SAIP) are evaluated using objective evaluation metrics (PSNR, SSIM) and subjective evaluation methods on real low-light traffic image datasets. The experimental results demonstrate the superiority, robustness, and effectiveness of the defogging + enhancement method for image defogging in low-light fog traffic environments. Object detection and recognition in low-light foggy traffic images are improved by the joint optimization learning method, outperforming advanced detection algorithms and non-joint methods. However, for low-light foggy environments, object detection techniques are highly correlated with fog depth and concentration estimation. The performance of the IDOD-YOLOV7 object detection algorithm can be further improved by introducing depth and fog concentration estimation.

## Figures and Tables

**Table 1:** The mapping functions of the three filters and the parameters to be optimized.

Filter	Mapping Function	Parameters
WB	$P_{o}=\left(W_{r}r_{i},W_{g}g_{i},W_{b}b_{i}\right)$	$W_{r},W_{g},W_{b}$
Gamma	$P_{o}=P_{i}^{G}$	G
Contrast	$P_{o}=\alpha \cdot En\left(P_{i}\right)+(1-\alpha )\cdot P_{i}$	α

**Table 2:** Quantitative evaluations on the benchmark dehazing datasets. ↑and↓mean the better methods should achieve higher/lower score of this metric.

Methods		DCP	EPDN	Dehazenet	AODNET	MSCNN	FFANET	PSD	CycleGan	YOLY	Ours
SOTS	PSNR↑	13.23	25.06	18.80	21.14	17.57	25.29	15.32	20.55	15.57	24.62
	SSIM↑	0.809	0.931	0.834	0.864	0.811	0.924	0.805	0.875	0.837	0.837
	CIEDE↓	7.508	4.578	5.632	4.325	6.328	2.347	13.24	7.547	14.216	4.295
HAZE	PSNR↑	13.10	15.02	13.23	18.68	14.67	12.00	15.30	15.29	14.74	18.87
	SSIM↑	0.699	0.763	0.833	0.843	0.796	0.592	0.800	0.756	0.688	0.845
	CIEDE↓	19.04	14.96	13.33	13.67	14.27	20.33	14.84	19.50	15.24	13.27
FTOD	PSNR↑	11.34	21.37	11.39	20.38	17.21	14.23	16.89	15.83	15.67	19.17
	SSIM↑	0.727	0.821	0.845	0.851	0.807	0.681	0.841	0.781	0.771	0.873
	CIEDE↓	16.73	14.05	14.78	13.57	14.37	17.21	13.87	18.17	17.26	13.21

**Table 3:** The mAP comparison on the different object detection methods at three fog concentrations.

Training Set	Test Set	Fast R-CNN	Faster R-CNN	SSD	YOLOV3	YOLOV7	IDOD-YOLOV7
Heavy	Heavy	0.5137	0.5316	0.5734	0.5048	0.5387	0.6036
Medium	Medium	0.5843	0.6217	0.6647	0.5916	0.6867	0.6917
Light	Light	0.6472	0.6618	0.6918	0.6264	0.7114	0.7304

**Table 4:** The mAP comparison on the different “dehazing + detection” methods at three fog concentrations and multiple haze level. (Y: YOLOV7).

Training Set	Test Set	AOD-NET + Y	DCP + Y	MSCNN + Y	Dehazenet + Y	Ours
Heavy	Heavy	0.5436	0.5612	0.5834	0.5448	0.6036
Medium	Medium	0.6983	0.6911	0.6847	0.6816	0.6917
Light	Light	0.7077	0.7051	0.7118	0.7164	0.7304
Multiple Haze Level	Heavy	-	-	-	-	0.6514
	Medium	-	-	-	-	0.6826
	Light	-	-	-	-	0.7143
	Multiple Haze Level					0.6786

**Table 5:** Ablation analysis on the low-light foggy in Image Dehazing Model (mAP).

Training Set	Model	Enhancement	Defog	Detection	Heavy	Medium	Light
Multiple Haze Level	YOLOV7			✔	0.5187	0.6467	0.6514
	Enhancement + YOLOV7	✔		✔	0.5217	0.6671	0.6872
	Defog + YOLOV7		✔	✔	0.5143	0.6481	0.6816
	IDOD-YOLOV7	✔	✔	✔	0.6514	0.6826	0.7143

**Table 6:** Complexity analysis of different object detection algorithms.

	Fast R-CNN	Faster R-CNN	SSD	YOLOV3	YOLOV7	IDOD-YOLOV7
FLOPs	13G	28G	71G	102G	89.7G	93.6G
#Param	7.4M	12.9M	0.6647M	62M	36.9M	46.5M
FPS	0.5	7	21	54	65	71

## Data Availability

Not applicable.
